# Supplementary material for: MIAMI-AD (Methylation in Aging and Methylation in AD): an integrative knowledgebase that facilitates explorations of DNA methylation across sex, aging, and Alzheimer’s disease
Source: Database (Oxford). 2024 Jul 19;2024:baae061. doi: 10.1093/database/baae061 (PMC11259044; doi:10.1093/database/baae061)
Supplement: baae061_Supp [file baae061_supp.zip › suppl_data/_ALL_supp-figures_5-6-2024.pdf]

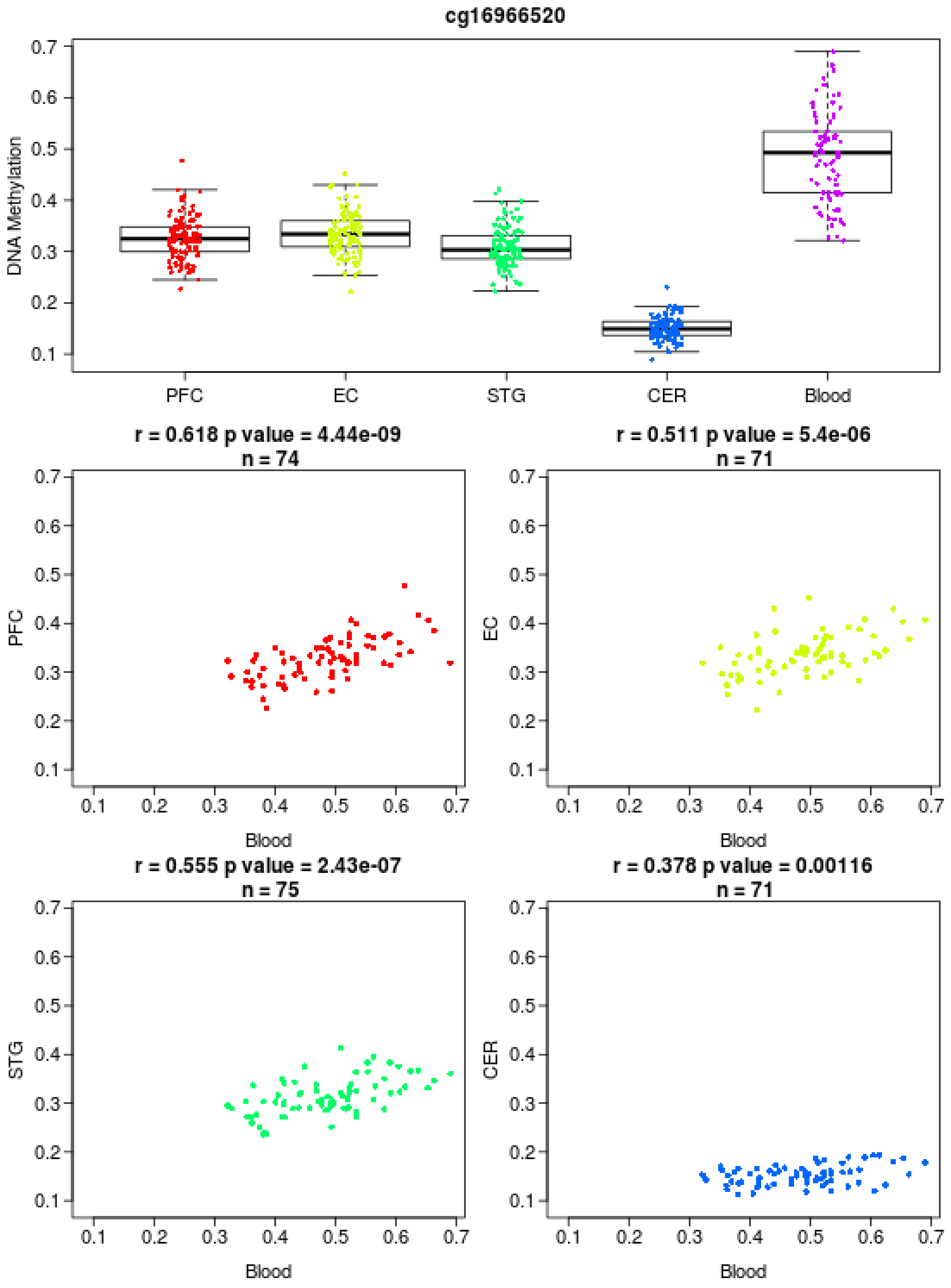


**Supplementary Figure 1** Blood DNA methylation at cg16966520 in the promoter region of the *EIF2D* gene is significantly associated with brain DNA methylation in the prefrontal cortex (PFC), entorhinal cortex (EC), superior temporal gyrus (STC), and cerebellum (CER). These results were obtained from the Blood Brain DNA Methylation Comparison Tool (https://epigenetics.essex.ac.uk/bloodbrain/).

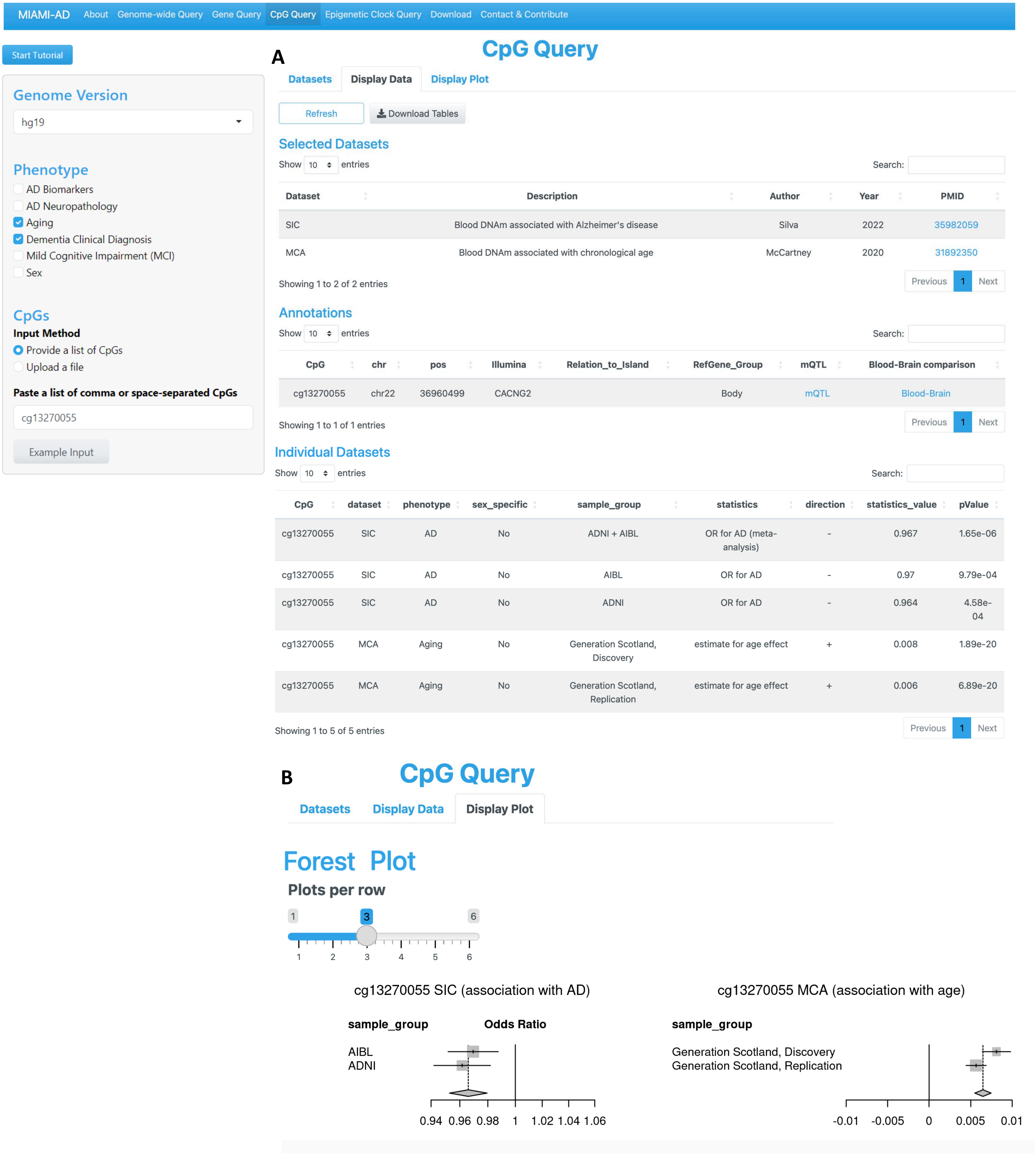


**Supplementary Figure 2** DNA methylation at cg13270055 located on the *CACNG2* gene increases with age ($P_{discovery}$ = 1.89 ×$10^{-20}$, $P_{replication}$ = 6.89 ×$10^{-20}$), but decreases in the blood samples of Alzheimer's subjects ($P_{meta\text{-}analysis}$ = 1.65×$10^{-6}$, $P_{AIBL}$ = 9.79×$10^{-4}$, $P_{ADNI}$ = 4.58×$10^{-4}$). The summary statistics are shown under **(A)** Display Data tab, and the forest plots are shown under **(B)** Display Plot tab.

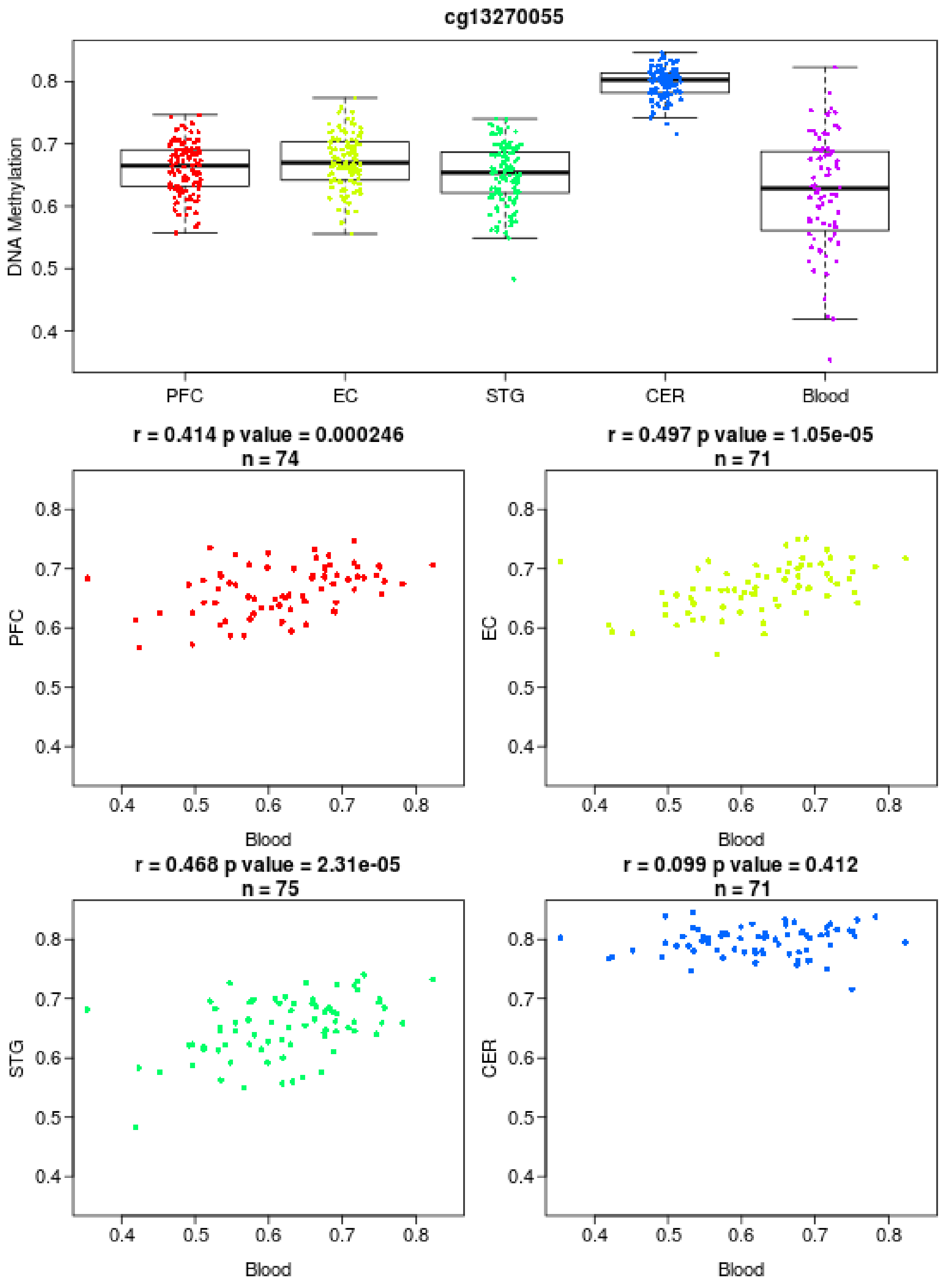


**Supplementary Figure 3** Blood DNA methylation at cg13270055 located on the *CACNG2* gene is significantly associated with brain DNA methylation in the prefrontal cortex (PFC), entorhinal cortex (EC), superior temporal gyrus (STC), and cerebellum (CER). These results were obtained from the Blood Brain DNA Methylation Comparison Tool (https://epigenetics.essex.ac.uk/bloodbrain/).

**A.** No CpG was selected when intersecting the female and male summary statistics dataset

## Genome-wide Query

Datasets | Display Data | Display Plot

Refresh | Download Tables

### Selected Datasets

**To select a different set of CpGs**

- change significance level: double click on **Threshold**

Show 10 entries Search:

| Dataset | Description | Author | Year | PMID | Source | Metric | Filter | Threshold |
|---|---|---|---|---|---|---|---|---|
| SIF | Blood DNAm associated with Alzheimer's disease in females | Silva | 2022 | 36109771 | ADNI + AIBL | ◉ pValue ○ FDR | ◉ < than ○ > than | 0.00001 |
| SIM | Blood DNAm associated with Alzheimer's disease in males | Silva | 2022 | 36109771 | ADNI + AIBL | ◉ pValue ○ FDR | ◉ < than ○ > than | 0.00001 |

Showing 1 to 2 of 2 entries Previous 1 Next

### Annotated CpGs

Explore Top 10 CpGs

Show 10 entries Search:

| CpG | chr | pos | Illumina | dataset | sample_group | phenotype | sex_specific | statistics | direction | statistics_value | pValue |
|---|---|---|---|---|---|---|---|---|---|---|---|
| No significant CpGs. - There are no CpGs that meet all of the threshold cutoffs. Please adjust the thresholds or the datasets selected. | | | | | | | | | | | |

Showing 0 to 0 of 0 entries Previous Next

**B.** Selecting female-specific CpGs associated with AD

Datasets | Display Data | Display Plot

Refresh | Download Tables

### Selected Datasets

**To select a different set of CpGs**

- change significance level: double click on **Threshold**

Show 10 entries Search:

| Dataset | Description | Author | Year | PMID | Source | Metric | Filter | Threshold |
|---|---|---|---|---|---|---|---|---|
| SIF | Blood DNAm associated with Alzheimer's disease in females | Silva | 2022 | 36109771 | ADNI + AIBL | ◉ pValue ○ FDR | ◉ < than ○ > than | 0.00001 |
| SIM | Blood DNAm associated with Alzheimer's disease in males | Silva | 2022 | 36109771 | ADNI + AIBL | ◉ pValue ○ FDR | ○ < than ◉ > than | 0.05 |

### Annotated CpGs

Explore Top 10 CpGs

Show 10 entries Search:

| CpG | chr | pos | Illumina | dataset | sample_group | phenotype | sex_specific | statistics | direction | statistics_value | pValue |
|---|---|---|---|---|---|---|---|---|---|---|---|
| cg18020072 | chr6 | 31590640 | SNORA38;BAT2 | SIF | ADNI + AIBL | AD | Yes, females | OR for AD (meta-analysis) | + | 1.328 | 3.02e-08 |
| cg18020072 | chr6 | 31590640 | SNORA38;BAT2 | SIM | ADNI + AIBL | AD | Yes, males | OR for AD (meta-analysis) | + | 1.051 | 2.49e-01 |
| cg24276069 | chr1 | 45243927 | RPS8;SNORD38B | SIF | ADNI + AIBL | AD | Yes, females | OR for AD (meta-analysis) | + | 1.233 | 9.62e-08 |
| cg24276069 | chr1 | 45243927 | RPS8;SNORD38B | SIM | ADNI + AIBL | AD | Yes, males | OR for AD (meta-analysis) | - | 0.955 | 2.08e-01 |

**C.** Selecting male-specific CpGs associated with AD

## Genome-wide Query

Datasets | Display Data | Display Plot

Refresh | Download Tables

### Selected Datasets

**To select a different set of CpGs**

- change significance level: double click on **Threshold**

Show 10 entries Search:

| Dataset | Description | Author | Year | PMID | Source | Metric | Filter | Threshold |
|---|---|---|---|---|---|---|---|---|
| SIF | Blood DNAm associated with Alzheimer's disease in females | Silva | 2022 | 36109771 | ADNI + AIBL | ◉ pValue ○ FDR | ○ < than ◉ > than | 0.05 |
| SIM | Blood DNAm associated with Alzheimer's disease in males | Silva | 2022 | 36109771 | ADNI + AIBL | ◉ pValue ○ FDR | ◉ < than ○ > than | 0.00001 |

### Annotated CpGs

Explore Top 10 CpGs

Show 10 entries Search:

| CpG | chr | pos | Illumina | dataset | sample_group | phenotype | sex_specific | statistics | direction | statistics_value | pValue |
|---|---|---|---|---|---|---|---|---|---|---|---|
| cg02672643 | chr11 | 110790527 | | SIF | ADNI + AIBL | AD | Yes, females | OR for AD (meta-analysis) | + | 1.049 | 2.05e-01 |
| cg02672643 | chr11 | 110790527 | | SIM | ADNI + AIBL | AD | Yes, males | OR for AD (meta-analysis) | + | 1.19 | 1.62e-06 |
| cg15757041 | chr16 | 5115938 | C16orf89 | SIF | ADNI + AIBL | AD | Yes, females | OR for AD (meta-analysis) | + | 1.058 | 4.70e-01 |
| cg15757041 | chr16 | 5115938 | C16orf89 | SIM | ADNI + AIBL | AD | Yes, males | OR for AD (meta-analysis) | - | 0.677 | 3.69e-06 |
| cg15281611 | chr1 | 54040884 | GLIS1 | SIF | ADNI + AIBL | AD | Yes, females | OR for AD (meta-analysis) | + | 1.058 | 1.07e-01 |
| cg15281611 | chr1 | 54040884 | GLIS1 | SIM | ADNI + AIBL | AD | Yes, males | OR for AD (meta-analysis) | + | 1.181 | 6.66e-06 |
| cg03827739 | chr10 | 75401754 | MYOZ1 | SIF | ADNI + AIBL | AD | Yes, females | OR for AD (meta-analysis) | + | 1.038 | 3.93e-01 |
| cg03827739 | chr10 | 75401754 | MYOZ1 | SIM | ADNI + AIBL | AD | Yes, males | OR for AD (meta-analysis) | + | 1.199 | 8.03e-06 |

**Supplementary Figure 4** Using Genome-wide Query to perform sex-specific analysis.

**A**. Selected Datasets and CpG

## CpG Query

Datasets | Display Data | Display Plot

Refresh | Download Tables

### Selected Datasets

Show 10 entries

Search:

| Dataset | Description | Author | Year | PMID |
|---|---|---|---|---|
| SIF | Blood DNAm associated with Alzheimer's disease in females | Silva | 2022 | 36109771 |
| SIM | Blood DNAm associated with Alzheimer's disease in males | Silva | 2022 | 36109771 |

Showing 1 to 2 of 2 entries

Previous 1 Next

### Annotations

Show 10 entries

Search:

| CpG | chr | pos | Illumina | Relation_to_Island | RefGene_Group | mQTL | Blood-Brain comparison |
|---|---|---|---|---|---|---|---|
| cg03546163 | chr6 | 35654363 | FKBP5;FKBP5;FKBP5;FKBP5 | N_Shore | 5'UTR;5'UTR;5'UTR;5'UTR | mQTL | Blood-Brain |

**B** Results under "Display Data" tab

### Individual Datasets

Show 10 entries

Search:

| CpG | dataset | sample_group | phenotype | sex_specific | statistics | direction | statistics_value | pValue |
|---|---|---|---|---|---|---|---|---|
| cg03546163 | SIF | ADNI + AIBL | AD | Yes, females | OR for AD (meta-analysis) | - | 0.911 | 1.23e-06 |
| cg03546163 | SIF | AIBL | AD | Yes, females | OR for AD | - | 0.896 | 8.86e-05 |
| cg03546163 | SIF | ADNI | AD | Yes, females | OR for AD | - | 0.924 | 3.02e-03 |
| cg03546163 | SIM | ADNI + AIBL | AD | Yes, males | OR for AD (meta-analysis) | - | 0.988 | 5.11e-01 |
| cg03546163 | SIM | AIBL | AD | Yes, males | OR for AD | - | 0.95 | 9.45e-02 |
| cg03546163 | SIM | ADNI | AD | Yes, males | OR for AD | + | 1.008 | 7.16e-01 |

**C** Results under the "Display Plot" tab

## CpG Query

Datasets | Display Data | Display Plot

Refresh

## Forest Plot

### Plots per row

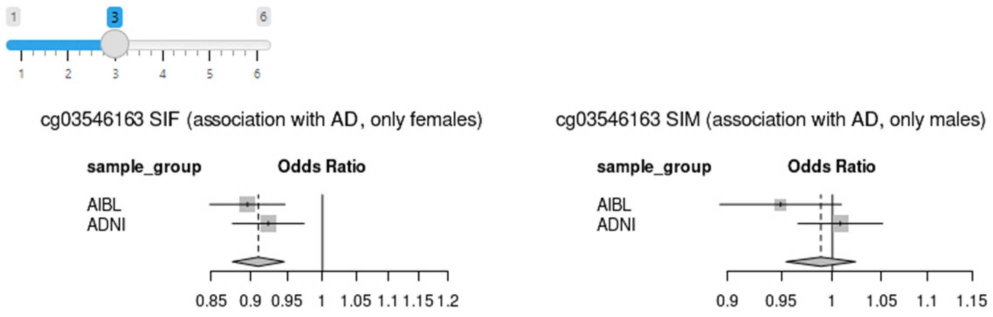


**Supplementary Figure 5** The CpG Query Tool can be used to explore details of DNA methylation at female-specific CpG cg03546163 associated with AD.
