# Supplementary material for: MIAMI-AD (Methylation in Aging and Methylation in AD): an integrative knowledgebase that facilitates explorations of DNA methylation across sex, aging, and Alzheimer’s disease
Source: Database (Oxford). 2024 Jul 19;2024:baae061. doi: 10.1093/database/baae061 (PMC11259044; doi:10.1093/database/baae061)
Supplement: baae061_Supp [file baae061_supp.zip › suppl_data/_All_supp-tables_5-7-2024.pdf]

Supplementary Table 1 Detailed information of studies included in MIAMI-AD database.

Phenotype	Tissue	Sex-specific	PMID	Title	First Author	Year	Journal	Open Access / PubMed Access	Included Tables	Number of Tables
clinical AD diagnosis	blood	N	35982059	Cross-tissue analysis of blood and brain epigenome-wide association studies in Alzheimer's disease	Silva	2022	Nat Commun.	Open Access	Table 1-3<br>Supp Data 2	4
clinical AD diagnosis	blood	Y	36109771	Distinct sex-specific DNA methylation differences in Alzheimer's disease	Silva	2022	Alzheimers Res. Ther.	Open Access	Table 2-3<br>Supp Table 2-6	7
clinical AD progression, conversion to MCI or AD	blood	N	34654479	Association of peripheral blood DNA methylation level with Alzheimer's disease progression	Li	2021	Clin Epigenetics	Open Access	Table S1	1
clinical AD diagnosis, conversion to MCI	blood	N	32745807	An epigenome-wide association study of Alzheimer's disease blood highlights robust DNA hypermethylation in the HOXB6 gene	Roubroeks	2020	Neurobiol Aging	Open Access	Table 2<br>Supp Table 1-5	6
dementia diagnosis	blood	N	32671182	Blood DNA methylation signatures to detect dementia prior to overt clinical symptoms	Fransquet	2020	Alzheimers Dement	Open Access	Table 2<br>Supp Table 3-4	3
AD neuropathology	brain PFC	N	33257653	Epigenome-wide meta-analysis of DNA methylation differences in prefrontal cortex implicates the immune processes in Alzheimer's disease	Zhang	2020	Nat Commun.	Open Access	Supp Data 2, 15	2
AD neuropathology	brain PFC	Y	33902726	Sex-specific DNA methylation changes in Alzheimer's disease pathology	Zhang	2021	Acta Neuropathol Commun	Open Access	Supp Table 3-6	4
AD neuropathology	brain PFC	N	25129075	Alzheimer's disease: early alterations in brain DNA methylation at ANK1, BIN1, RHBDF2 and other loci	De Jager	2014	Nat Neurosci	PubMed Access	Supp Table 2	1
AD neuropathology	brain PFC, EC, STG, CER	N	25129077	Methylomic profiling implicates cortical deregulation of ANK1 in Alzheimer's disease	Lunnon	2014	Nat Neurosci	PubMed Access	Supp Table 3-6	4
clinical AD diagnosis	blood	N	25129077	Methylomic profiling implicates cortical deregulation of ANK1 in Alzheimer's disease	Lunnon	2014	Nat Neurosci.	PubMed Access	Supp Table 12	1
AD neuropathology	brain PFC, STG, EC	N	34112773	A meta-analysis of epigenome-wide association studies in Alzheimer's disease highlights novel differentially methylated loci across cortex	Smith	2021	Nat Commun.	Open Access	Supp Data 1-6	6
AD neuropathology	brain PG	N	36811307	Genome-wide methylomic regulation of multiscale gene networks in Alzheimer's disease	Wang	2023	Alzheimers Dement.	Open Access	Supp Data 2a,c	2
AD neuropathology	brain FC, TC, EC, CER	N	33790779	A Meta-Analysis of Brain DNA Methylation Across Sex, Age, and Alzheimer's Disease Points for Accelerated Epigenetic Aging in Neurodegeneration	Pellegrini	2021	Front Aging Neurosci	Open Access	Supp File 1,3,5,6	4
sex	brain PFC	Y	30976086	Sex-differential DNA methylation and associated regulation networks in human brain implicated in the sex-biased risks of psychiatric disorders	Xia	2021	Mol Psychiatry	Open Access	Supp Table 2,3	2
AD neuropathology	brain EC	N	37149695	Entorhinal cortex epigenome-wide association study highlights four novel loci showing differential methylation in Alzheimer's disease	Sommerer	2023	Alzheimers Res. Ther.	Open Access	https://www.liga.uni-luebeck.de/sommerer_AD_entorhinal_cortex_EWAS_results/	1
clinical AD status	brain EC	N	37149695	Entorhinal cortex epigenome-wide association study highlights four novel loci showing differential methylation in Alzheimer's disease	Sommerer	2023	Alzheimers Res. Ther.	Open Access		1
AD neuropathology	brain IFG, STG	N	33069246	Epigenome-wide association study of Alzheimer's disease replicates 22 differentially methylated positions and 30 differentially methylated regions	Li	2020	Clin Epigenetics	Open Access	Supp Table 1, 2A, 2B	3
clinical AD status	brain MTG	N	36596913	Integrated DNA Methylation/RNA Profiling in Middle Temporal Gyrus of Alzheimer's Disease	Piras	2023	Cell Mol Neurobiol	Open Access	Supp Table 1,7	2
clinical AD status	brain MTG	Y	36596913	Integrated DNA Methylation/RNA Profiling in Middle Temporal Gyrus of Alzheimer's Disease	Piras	2023	Cell Mol Neurobiol	Open Access	Supp Table 3	1
AD neuropathology	brain MTG	N	36596913	Integrated DNA Methylation/RNA Profiling in Middle Temporal Gyrus of Alzheimer's Disease	Piras	2023	Cell Mol Neurobiol	Open Access	Supp Table 9.10	2
aging	blood	N	31892350	An epigenome-wide association study of sex-specific chronological ageing	McCartney	2020	Genome Med.	Open Access	Supp Table 1,2	2
sex	blood	Y	31892350	An epigenome-wide association study of sex-specific chronological ageing	McCartney	2020	Genome Med.	Open Access	Supp Table 3	1
aging, sex	blood	Y	31892350	An epigenome-wide association study of sex-specific chronological ageing	McCartney	2020	Genome Med.	Open Access	Supp Table 4,5	2
aging	blood	N	32147700	Blood DNA Methylation and Aging: A Cross-Sectional Analysis and Longitudinal Validation in the InCHIANTI Study	Tharakan	2020	J Gerontol A Biol Sci Med Sci	Open Access	Table 1<br>Supp Table 2	2
aging	blood	N	25404168	Age-related variations in the methylome associated with gene expression in human monocytes and T cells	Reynolds	2014	Nat Commun	Open Access	Supp Data 1	1
aging	blood	N	25701668	A meta-analysis on age-associated changes in blood DNA methylation: results from an original analysis pipeline for Infinium 450k data	Bacalini	2015	Aging	Open Access	Table 1	1
aging, sex	blood	Y	33276343	Age-related DNA methylation changes are sex-specific: a comprehensive assessment	Yusipov	2020	Aging	Open Access	Supp Table 1, 3	2
aging, epignetic clock	blood	N	31443728	Improved precision of epigenetic clock estimates across tissues and its implication for biological ageing	Zhang	2019	Genome Med.	Open Access	https://github.com/qzhang314/DNAm-based-age-predictor/blob/master/en.coef	1
aging	blood	N	30264654	Epigenetic influences on aging: a longitudinal genome-wide methylation study in old Swedish twins	Wang	2018	Epigenetics	Open Access	Supp Table 1	1
aging	blood	N	25888029	Ageing-associated changes in the human DNA methylome: genomic locations and effects on gene expression	Marttila	2015	BMC Genomics	Open Access	Supp Table 1	1
sex	blood	N	25888029	Ageing-associated changes in the human DNA methylome: genomic locations and effects on gene expression	Marttila	2015	BMC Genomics	Open Access	Supp Table 9	1
sex	Saliva	Y	37300819	Sex-specific DNA methylation in saliva from the multi-ethnic Future of Families and Child Wellbeing Study	Reiner	2023	Epigenetics	Open Access	Supp Table 2	1
biomarker	blood	N	37038196	Distinct CSF biomarker-associated DNA methylation in Alzheimer's disease and cognitively normal subjects	Zhang	2023	Alzheimers Res. Ther.	Open Access	Table 2-5<br>Supp Table 4-9	10
aging, epignetic clock	blood	N	35029144	DunedinPACE, a DNA methylation biomarker of the pace of aging	Belsky	2022	Elife	Open Access	Supp File 1B	1
aging, epignetic clock	blood	Y	33978960	Genome-wide identification of age-related CpG sites for age estimation from blood DNA of Han Chinese individuals	Xiao	2021	Aging	Self Archiving policy*	Supp Table 3-5	3
aging, epignetic clock	blood	N	32367804	Quantification of the pace of biological aging in humans through a blood test, the DunedinPoAm DNA methylation algorithm	Belsky	2020	Elife	Open Access	Supp File 1A	1
aging, epignetic clock	brain cortex	N	33300551	Recalibrating the epigenetic clock: implications for assessing biological age in the human cortex	Shireby	2020	Brain	Open Access	https://github.com/gemmashireby/CorticalClock	1
aging, epignetic clock	human fibroblasts, keratinocytes, buccal cells, endothelial cells, blood, and saliva	N	30048243	Epigenetic clock for skin and blood cells applied to Hutchinson Gilford Progeria Syndrome and ex vivo studies	Horvath	2018	Aging	Open Access	Supp Data 2	1
aging, epignetic clock	blood	N	29676998	An epigenetic biomarker of aging for lifespan and healthspan	Levine	2018	Aging	Open Access	Supp File 2	1
aging, epignetic clock	blood	N	28303888	DNA methylation signatures in peripheral blood strongly predict all-cause mortality	Zhang	2017	Nat Commun.	Open Access	Supp Fig 1	1
aging, epignetic clock	blood	N	26928272	DNA methylation levels at individual age-associated CpG sites can be indicative for life expectancy	Lin	2016	Aging	Open Access	Supp Table 1	1
aging, epignetic clock	blood	N	27471517	Simplified assay for epigenetic age estimation in whole blood of adults	Vidal-Bralo	2016	Front Genet	Open Access	Table 2	1
aging, epignetic clock	blood	N	23177740	Genome-wide Methylation Profiles Reveal Quantitative Views of Human Aging Rates	Hannum	2013	Mol. Cell.	Open Access	Supp 2	1
aging, epignetic clock	51 tissue and cell types	N	24138928	DNA methylation age of human tissues and cell types	Horvath	2013	Genome Biol.	Open Access	Supp File 3	1
aging, epignetic clock	Saliva	N	21731603	Epigenetic predictor of age	Bocklandt	2011	PLoS One	Open Access	Supp Table 1-2	2
									Total	**98**

Abbreviations

CER: cerebellum
EC: entorhinal cortex
MTG: middle temporal gyrus
PFC: prefrontal cortex
PG: parahippocampal gyrus
STG: superior temporal gyrus
FC: frontal cortex
IFG: inferior frontal gyrus
TC: temporal cortex

* See details at https://authorservices.wiley.com/author-resources/Journal-Authors/licensing/self-archiving.html

Supplmentary Table 2 Using Genome-wide Query Tool, we obtained a total of 27 CpGs reaching 5% FDR in both discovery and replication analyses in aging (McCartney et al. 2020, PMID: 31892350) and with a meta-analysis P-value < 10^{-5} or less in AD (Silva et al. 2022, PMID: 35982059). These results were downloaded using the "Download Tables" button under "Display Data".

CpG	chr	pos	Illumina	dataset	sample_group	phenotype	sex_specific	statistics	direction	statistics_value	pValue
cg06968164	chr13	45629698		SIC	ADNI + AIBL	AD	No	OR for AD (meta-analysis)	-	0.929	9.64E-06
cg06968164	chr13	45629698		MCA	Generation Scotland, Discovery	Aging	No	estimate for age effect	-	-0.002	4.13E-10
cg06968164	chr13	45629698		MCA	Generation Scotland, Replication	Aging	No	estimate for age effect	-	-0.007	1.01E-291
cg00848167	chr15	89921898	LOC254559	SIC	ADNI + AIBL	AD	No	OR for AD (meta-analysis)	-	0.894	1.04E-06
cg00848167	chr15	89921898	LOC254559	MCA	Generation Scotland, Discovery	Aging	No	estimate for age effect	+	0.013	3.04E-145
cg00848167	chr15	89921898	LOC254559	MCA	Generation Scotland, Replication	Aging	No	estimate for age effect	+	0.01	2.34E-206
cg21821767	chr17	9769170	GLP2R	SIC	ADNI + AIBL	AD	No	OR for AD (meta-analysis)	-	0.919	5.00E-06
cg21821767	chr17	9769170	GLP2R	MCA	Generation Scotland, Discovery	Aging	No	estimate for age effect	-	-0.006	1.30E-51
cg21821767	chr17	9769170	GLP2R	MCA	Generation Scotland, Replication	Aging	No	estimate for age effect	-	-0.007	4.91E-159
cg10875957	chr6	41122461	TREML1	SIC	ADNI + AIBL	AD	No	OR for AD (meta-analysis)	-	0.899	2.78E-06
cg10875957	chr6	41122461	TREML1	MCA	Generation Scotland, Discovery	Aging	No	estimate for age effect	-	-0.002	5.50E-11
cg10875957	chr6	41122461	TREML1	MCA	Generation Scotland, Replication	Aging	No	estimate for age effect	-	-0.004	2.74E-130
cg19459094	chr5	176856845	GRK6	SIC	ADNI + AIBL	AD	No	OR for AD (meta-analysis)	-	0.837	5.13E-06
cg19459094	chr5	176856845	GRK6	MCA	Generation Scotland, Discovery	Aging	No	estimate for age effect	-	-0.001	2.44E-05
cg19459094	chr5	176856845	GRK6	MCA	Generation Scotland, Replication	Aging	No	estimate for age effect	-	-0.004	8.04E-97
cg10022349	chr14	75041541	LTBP2	SIC	ADNI + AIBL	AD	No	OR for AD (meta-analysis)	+	1.108	9.69E-06
cg10022349	chr14	75041541	LTBP2	MCA	Generation Scotland, Discovery	Aging	No	estimate for age effect	+	0.002	5.91E-14
cg10022349	chr14	75041541	LTBP2	MCA	Generation Scotland, Replication	Aging	No	estimate for age effect	+	0.002	6.64E-44
cg16966520	chr1	206786174	LGTN	SIC	ADNI + AIBL	AD	No	OR for AD (meta-analysis)	+	1.047	3.45E-06
cg16966520	chr1	206786174	LGTN	MCA	Generation Scotland, Discovery	Aging	No	estimate for age effect	+	0.005	2.90E-21
cg16966520	chr1	206786174	LGTN	MCA	Generation Scotland, Replication	Aging	No	estimate for age effect	+	0.005	1.49E-40
cg22883080	chr22	36841223		SIC	ADNI + AIBL	AD	No	OR for AD (meta-analysis)	+	1.112	5.19E-06
cg22883080	chr22	36841223		MCA	Generation Scotland, Discovery	Aging	No	estimate for age effect	+	0.001	5.00E-04
cg22883080	chr22	36841223		MCA	Generation Scotland, Replication	Aging	No	estimate for age effect	+	0.002	4.21E-38
cg11920110	chr1	33926658		SIC	ADNI + AIBL	AD	No	OR for AD (meta-analysis)	+	1.109	6.07E-07
cg11920110	chr1	33926658		MCA	Generation Scotland, Discovery	Aging	No	estimate for age effect	+	0.003	1.09E-24
cg11920110	chr1	33926658		MCA	Generation Scotland, Replication	Aging	No	estimate for age effect	+	0.002	2.95E-29
cg14195992	chr8	48265917	KIAA0146	SIC	ADNI + AIBL	AD	No	OR for AD (meta-analysis)	-	0.874	1.43E-07
cg14195992	chr8	48265917	KIAA0146	MCA	Generation Scotland, Discovery	Aging	No	estimate for age effect	+	0.001	4.35E-05
cg14195992	chr8	48265917	KIAA0146	MCA	Generation Scotland, Replication	Aging	No	estimate for age effect	+	0.002	8.02E-25
cg13270055	chr22	36960499	CACNG2	SIC	ADNI + AIBL	AD	No	OR for AD (meta-analysis)	-	0.967	1.65E-06
cg13270055	chr22	36960499	CACNG2	MCA	Generation Scotland, Discovery	Aging	No	estimate for age effect	+	0.008	1.89E-20
cg13270055	chr22	36960499	CACNG2	MCA	Generation Scotland, Replication	Aging	No	estimate for age effect	+	0.006	6.89E-20
cg17317003	chr7	2728884	AMZ1	SIC	ADNI + AIBL	AD	No	OR for AD (meta-analysis)	-	0.938	7.68E-06
cg17317003	chr7	2728884	AMZ1	MCA	Generation Scotland, Discovery	Aging	No	estimate for age effect	-	-0.002	4.50E-03
cg17317003	chr7	2728884	AMZ1	MCA	Generation Scotland, Replication	Aging	No	estimate for age effect	-	-0.004	1.95E-18
cg17612535	chr5	932900		SIC	ADNI + AIBL	AD	No	OR for AD (meta-analysis)	-	0.91	7.04E-06
cg17612535	chr5	932900		MCA	Generation Scotland, Discovery	Aging	No	estimate for age effect	-	-0.001	6.75E-05
cg17612535	chr5	932900		MCA	Generation Scotland, Replication	Aging	No	estimate for age effect	-	-0.001	7.56E-18
cg22028323	chr10	98486758		SIC	ADNI + AIBL	AD	No	OR for AD (meta-analysis)	+	1.087	5.23E-06
cg22028323	chr10	98486758		MCA	Generation Scotland, Discovery	Aging	No	estimate for age effect	+	0.003	9.11E-18
cg22028323	chr10	98486758		MCA	Generation Scotland, Replication	Aging	No	estimate for age effect	+	0.001	2.35E-08
cg10855395	chr12	125413124		SIC	ADNI + AIBL	AD	No	OR for AD (meta-analysis)	-	0.954	4.59E-06
cg10855395	chr12	125413124		MCA	Generation Scotland, Discovery	Aging	No	estimate for age effect	+	0.004	2.04E-12
cg10855395	chr12	125413124		MCA	Generation Scotland, Replication	Aging	No	estimate for age effect	+	0.002	1.03E-09
cg07886485	chr3	58318093	PXK	SIC	ADNI + AIBL	AD	No	OR for AD (meta-analysis)	-	0.935	6.08E-06
cg07886485	chr3	58318093	PXK	MCA	Generation Scotland, Discovery	Aging	No	estimate for age effect	+	0.001	1.61E-04
cg07886485	chr3	58318093	PXK	MCA	Generation Scotland, Replication	Aging	No	estimate for age effect	-	-0.002	1.13E-11
cg10213353	chr20	60860905	OSBPL2	SIC	ADNI + AIBL	AD	No	OR for AD (meta-analysis)	-	0.923	3.23E-06
cg10213353	chr20	60860905	OSBPL2	MCA	Generation Scotland, Discovery	Aging	No	estimate for age effect	+	0.002	6.69E-10
cg10213353	chr20	60860905	OSBPL2	MCA	Generation Scotland, Replication	Aging	No	estimate for age effect	+	0.001	1.06E-02
cg09862283	chr1	1256375	CPSF3L	SIC	ADNI + AIBL	AD	No	OR for AD (meta-analysis)	-	0.827	8.39E-06
cg09862283	chr1	1256375	CPSF3L	MCA	Generation Scotland, Discovery	Aging	No	estimate for age effect	-	-0.001	4.05E-05
cg09862283	chr1	1256375	CPSF3L	MCA	Generation Scotland, Replication	Aging	No	estimate for age effect	+	0.001	3.22E-09
cg02672643	chr11	110790527		SIC	ADNI + AIBL	AD	No	OR for AD (meta-analysis)	+	1.111	3.89E-06
cg02672643	chr11	110790527		MCA	Generation Scotland, Discovery	Aging	No	estimate for age effect	+	0.002	1.69E-08
cg02672643	chr11	110790527		MCA	Generation Scotland, Replication	Aging	No	estimate for age effect	-	-0.001	4.75E-06
cg25578124	chr9	124014937		SIC	ADNI + AIBL	AD	No	OR for AD (meta-analysis)	+	1.116	1.20E-06
cg25578124	chr9	124014937		MCA	Generation Scotland, Discovery	Aging	No	estimate for age effect	+	0.001	3.61E-08
cg25578124	chr9	124014937		MCA	Generation Scotland, Replication	Aging	No	estimate for age effect	+	0.001	1.74E-07
cg25114611	chr6	35696870	KBP5;LOC28584	SIC	ADNI + AIBL	AD	No	OR for AD (meta-analysis)	-	0.909	3.29E-06
cg25114611	chr6	35696870	KBP5;LOC28584	MCA	Generation Scotland, Discovery	Aging	No	estimate for age effect	+	0.001	1.24E-02
cg25114611	chr6	35696870	KBP5;LOC28584	MCA	Generation Scotland, Replication	Aging	No	estimate for age effect	-	-0.001	5.54E-07
cg09402485	chr2	79924440	CTNNA2	SIC	ADNI + AIBL	AD	No	OR for AD (meta-analysis)	-	0.943	2.32E-06
cg09402485	chr2	79924440	CTNNA2	MCA	Generation Scotland, Discovery	Aging	No	estimate for age effect	-	-0.002	3.81E-03
cg09402485	chr2	79924440	CTNNA2	MCA	Generation Scotland, Replication	Aging	No	estimate for age effect	-	-0.002	8.02E-07
cg18262682	chr13	21578733	LATS2	SIC	ADNI + AIBL	AD	No	OR for AD (meta-analysis)	-	0.92	1.49E-06
cg18262682	chr13	21578733	LATS2	MCA	Generation Scotland, Discovery	Aging	No	estimate for age effect	+	0.002	7.76E-06
cg18262682	chr13	21578733	LATS2	MCA	Generation Scotland, Replication	Aging	No	estimate for age effect	+	0.001	1.57E-03
cg15448385	chr19	40903642	PRX	SIC	ADNI + AIBL	AD	No	OR for AD (meta-analysis)	-	0.879	2.10E-06
cg15448385	chr19	40903642	PRX	MCA	Generation Scotland, Discovery	Aging	No	estimate for age effect	-	-0.001	1.37E-04
cg15448385	chr19	40903642	PRX	MCA	Generation Scotland, Replication	Aging	No	estimate for age effect	+	0.001	4.31E-03
cg03763444	chr22	37753688	ELFN2	SIC	ADNI + AIBL	AD	No	OR for AD (meta-analysis)	+	1.121	9.79E-06
cg03763444	chr22	37753688	ELFN2	MCA	Generation Scotland, Discovery	Aging	No	estimate for age effect	-	-0.001	2.63E-06
cg03763444	chr22	37753688	ELFN2	MCA	Generation Scotland, Replication	Aging	No	estimate for age effect	-	0	4.66E-03
cg21881364	chr11	72438004	ARAP1	SIC	ADNI + AIBL	AD	No	OR for AD (meta-analysis)	-	0.816	5.64E-06
cg21881364	chr11	72438004	ARAP1	MCA	Generation Scotland, Discovery	Aging	No	estimate for age effect	-	-0.002	2.36E-05
cg21881364	chr11	72438004	ARAP1	MCA	Generation Scotland, Replication	Aging	No	estimate for age effect	+	0.001	3.50E-02
cg09698960	chr15	91498962	RCCD1	SIC	ADNI + AIBL	AD	No	OR for AD (meta-analysis)	-	0.954	8.09E-06
cg09698960	chr15	91498962	RCCD1	MCA	Generation Scotland, Discovery	Aging	No	estimate for age effect	-	-0.003	1.89E-05
cg09698960	chr15	91498962	RCCD1	MCA	Generation Scotland, Replication	Aging	No	estimate for age effect	-	-0.002	1.78E-03

Supplementary Table 3 Female-specific CpGs associated with AD in Silva et al. (2022) (PMID: 36109771). Using Genome-wide Query Tool, we obtained 21 CpGs with *P*- value < 10^{-5} or less in females, but with *P*- value > 0.05 in males. These results were downloaded using the "Download Tables" button under "Display Data".

CpG	chr	pos	Illumina	dataset	sample_group	phenotype	sex_specific	statistics	direction	statistics_value	pValue
cg18020072	chr6	31590640	SNORA38;BAT2	SIF	ADNI + AIBL	AD	Yes, females	OR for AD (meta-analysis)	+	1.328	3.02E-08
cg18020072	chr6	31590640	SNORA38;BAT2	SIM	ADNI + AIBL	AD	Yes, males	OR for AD (meta-analysis)	+	1.051	2.49E-01
cg24276069	chr1	45243927	RPS8;SNORD38B	SIF	ADNI + AIBL	AD	Yes, females	OR for AD (meta-analysis)	+	1.233	9.62E-08
cg24276069	chr1	45243927	RPS8;SNORD38B	SIM	ADNI + AIBL	AD	Yes, males	OR for AD (meta-analysis)	-	0.955	2.08E-01
cg01101459	chr1	234871477		SIF	ADNI + AIBL	AD	Yes, females	OR for AD (meta-analysis)	+	1.218	3.88E-07
cg01101459	chr1	234871477		SIM	ADNI + AIBL	AD	Yes, males	OR for AD (meta-analysis)	+	1.017	6.20E-01
cg03546163	chr6	35654363	FKBP5	SIF	ADNI + AIBL	AD	Yes, females	OR for AD (meta-analysis)	-	0.911	1.23E-06
cg03546163	chr6	35654363	FKBP5	SIM	ADNI + AIBL	AD	Yes, males	OR for AD (meta-analysis)	-	0.988	5.11E-01
cg16005224	chr12	101603174	SLC5A8	SIF	ADNI + AIBL	AD	Yes, females	OR for AD (meta-analysis)	+	1.071	1.69E-06
cg16005224	chr12	101603174	SLC5A8	SIM	ADNI + AIBL	AD	Yes, males	OR for AD (meta-analysis)	+	1.008	5.70E-01
cg19620629	chr10	132007272		SIF	ADNI + AIBL	AD	Yes, females	OR for AD (meta-analysis)	+	1.275	1.70E-06
cg19620629	chr10	132007272		SIM	ADNI + AIBL	AD	Yes, males	OR for AD (meta-analysis)	-	0.968	3.80E-01
cg14727962	chr5	178784230		SIF	ADNI + AIBL	AD	Yes, females	OR for AD (meta-analysis)	-	0.839	1.93E-06
cg14727962	chr5	178784230		SIM	ADNI + AIBL	AD	Yes, males	OR for AD (meta-analysis)	-	0.966	2.32E-01
cg24147779	chr15	91094168	CRTC3	SIF	ADNI + AIBL	AD	Yes, females	OR for AD (meta-analysis)	+	1.145	1.97E-06
cg24147779	chr15	91094168	CRTC3	SIM	ADNI + AIBL	AD	Yes, males	OR for AD (meta-analysis)	+	1.036	1.72E-01
cg27216853	chr2	10205672	CYS1	SIF	ADNI + AIBL	AD	Yes, females	OR for AD (meta-analysis)	+	1.174	2.04E-06
cg27216853	chr2	10205672	CYS1	SIM	ADNI + AIBL	AD	Yes, males	OR for AD (meta-analysis)	+	1.038	2.43E-01
cg25720825	chr1	2849682		SIF	ADNI + AIBL	AD	Yes, females	OR for AD (meta-analysis)	-	0.895	2.30E-06
cg25720825	chr1	2849682		SIM	ADNI + AIBL	AD	Yes, males	OR for AD (meta-analysis)	-	0.958	8.74E-02
cg07091793	chr10	6362483		SIF	ADNI + AIBL	AD	Yes, females	OR for AD (meta-analysis)	+	1.127	2.70E-06
cg07091793	chr10	6362483		SIM	ADNI + AIBL	AD	Yes, males	OR for AD (meta-analysis)	+	1.027	3.13E-01
cg02354658	chr5	95151391	GLRX	SIF	ADNI + AIBL	AD	Yes, females	OR for AD (meta-analysis)	+	1.114	3.18E-06
cg02354658	chr5	95151391	GLRX	SIM	ADNI + AIBL	AD	Yes, males	OR for AD (meta-analysis)	+	1.008	6.88E-01
cg01995927	chr1	24257839		SIF	ADNI + AIBL	AD	Yes, females	OR for AD (meta-analysis)	+	1.144	3.23E-06
cg01995927	chr1	24257839		SIM	ADNI + AIBL	AD	Yes, males	OR for AD (meta-analysis)	-	0.992	7.72E-01
cg15999041	chr17	28699933		SIF	ADNI + AIBL	AD	Yes, females	OR for AD (meta-analysis)	+	1.187	3.61E-06
cg15999041	chr17	28699933		SIM	ADNI + AIBL	AD	Yes, males	OR for AD (meta-analysis)	-	0.988	7.31E-01
cg26995819	chr19	46902695		SIF	ADNI + AIBL	AD	Yes, females	OR for AD (meta-analysis)	-	0.935	4.37E-06
cg26995819	chr19	46902695		SIM	ADNI + AIBL	AD	Yes, males	OR for AD (meta-analysis)	-	0.991	5.52E-01
cg01004980	chr3	48883069	PRKAR2A	SIF	ADNI + AIBL	AD	Yes, females	OR for AD (meta-analysis)	+	1.173	4.64E-06
cg01004980	chr3	48883069	PRKAR2A	SIM	ADNI + AIBL	AD	Yes, males	OR for AD (meta-analysis)	+	1.008	8.06E-01
cg24843003	chr19	1409547	DAZAP1	SIF	ADNI + AIBL	AD	Yes, females	OR for AD (meta-analysis)	+	1.197	5.51E-06
cg24843003	chr19	1409547	DAZAP1	SIM	ADNI + AIBL	AD	Yes, males	OR for AD (meta-analysis)	+	1.023	5.16E-01
cg19054768	chr19	53194874	ZNF83	SIF	ADNI + AIBL	AD	Yes, females	OR for AD (meta-analysis)	+	1.059	5.89E-06
cg19054768	chr19	53194874	ZNF83	SIM	ADNI + AIBL	AD	Yes, males	OR for AD (meta-analysis)	+	1.004	7.05E-01
cg04894981	chr3	5114456		SIF	ADNI + AIBL	AD	Yes, females	OR for AD (meta-analysis)	+	1.202	7.92E-06
cg04894981	chr3	5114456		SIM	ADNI + AIBL	AD	Yes, males	OR for AD (meta-analysis)	+	1.037	3.61E-01
cg02314501	chr3	14639595		SIF	ADNI + AIBL	AD	Yes, females	OR for AD (meta-analysis)	+	1.086	8.34E-06
cg02314501	chr3	14639595		SIM	ADNI + AIBL	AD	Yes, males	OR for AD (meta-analysis)	+	1.011	5.51E-01
cg14528056	chr1	155194782	GBAP1	SIF	ADNI + AIBL	AD	Yes, females	OR for AD (meta-analysis)	+	1.164	9.11E-06
cg14528056	chr1	155194782	GBAP1	SIM	ADNI + AIBL	AD	Yes, males	OR for AD (meta-analysis)	+	1.019	5.64E-01

Supplementary Table 4 Male-specific CpGs associated with AD in Silva et al. (2022) (PMID: 36109771). Using Genome-wide Query Tool, we obtained 4 CpGs with *P*- value < 10^{-5} or less in males, but with *P*- value > 0.05 in females. These results were downloaded using the "Download Tables" button under "Display Data".

CpG	chr	pos	Illumina	dataset	sample_group	phenotype	sex_specific	statistics	direction	statistics_value	pValue
cg02672643	chr11	110790527		SIF	ADNI + AIBL	AD	Yes, females	OR for AD (meta-analysis)	+	1.049	2.05E-01
cg02672643	chr11	110790527		SIM	ADNI + AIBL	AD	Yes, males	OR for AD (meta-analysis)	+	1.19	1.62E-06
cg15757041	chr16	5115938	C16orf89	SIF	ADNI + AIBL	AD	Yes, females	OR for AD (meta-analysis)	+	1.058	4.70E-01
cg15757041	chr16	5115938	C16orf89	SIM	ADNI + AIBL	AD	Yes, males	OR for AD (meta-analysis)	-	0.677	3.69E-06
cg15281611	chr1	54040884	GLIS1	SIF	ADNI + AIBL	AD	Yes, females	OR for AD (meta-analysis)	+	1.058	1.07E-01
cg15281611	chr1	54040884	GLIS1	SIM	ADNI + AIBL	AD	Yes, males	OR for AD (meta-analysis)	+	1.181	6.66E-06
cg03827739	chr10	75401754	MYOZ1	SIF	ADNI + AIBL	AD	Yes, females	OR for AD (meta-analysis)	+	1.038	3.93E-01
cg03827739	chr10	75401754	MYOZ1	SIM	ADNI + AIBL	AD	Yes, males	OR for AD (meta-analysis)	+	1.199	8.03E-06

Supplementary Table 5 Independent validations of MIAMI-AD database by three co-authors (HH, WZ, DOS).

Phenotype	Sex-specific	PMID	Title	First Author	Year	Journal	Included Tables	Number of Tables	CpGs Tested in Validation	Genes/DMRs Tested in Validation	Reviewer
clinical AD diagnosis	N	35982059	Cross-tissue analysis of blood and brain epigenome-wide association studies in Alzheimer's disease	Silva	2022	Nat Commun.	Table 1-3<br>Supp Data 2	4	cg14195992,cg14727962,cg03718411,cg14103343,cg18137450,cg00682096,cg25578124,cg18262682	chr20:36148672-36148861; NNAT2<br>chr14:56777451-56777526; PELI2	HH
clinical AD diagnosis	N	35982059	Cross-tissue analysis of blood and brain epigenome-wide association studies in Alzheimer's disease	Silva	2022	Nat Commun.	Table 1-3<br>Supp Data 2	4	cg03429569, cg10570276, cg14727962, cg03718411, cg25840926, cg12234455, cg14019523, cg14622996	chr1:205819345-205819464;chr20:36149081-36149232;NNAT, XPO4	DOS
clinical AD diagnosis	N	35982059	Cross-tissue analysis of blood and brain epigenome-wide association studies in Alzheimer's disease	Silva	2022	Nat Commun.	Table 1-3<br>Supp Data 2	4	cg17612535 cg17928676 cg17317003 cg09698960 cg09862283 cg21500174 cg06357748 cg06968164 cg10022349 cg03763444	PM20D1 C1orf65	WZ
clinical AD diagnosis	Y	36109771	Distinct sex-specific DNA methylation differences in Alzheimer's disease	Silva	2022	Alzheimers Res. Ther.	Table 2-3<br>Supp Table 2	7	cg18020072,cg24276069,cg03546163,cg15757041,cg07697276,cg03407747,cg07533224,cg15713546	chr11:67383534-67384040; NUDT8,NDUFV1<br>chr3:111717793-111718245; TAGLN3<br>chr11:32417790-32417924; WT1	HH
clinical AD diagnosis	Y	36109771	Distinct sex-specific DNA methylation differences in Alzheimer's disease	Silva	2022	Alzheimers Res. Ther.	Table 2-3<br>Supp Table 2	7	cg02094419, cg06236312, cg06464775		DOS
clinical AD diagnosis	Y	36109771	Distinct sex-specific DNA methylation differences in Alzheimer's disease	Silva	2022	Alzheimers Res. Ther.	Table 2-3<br>Supp Table 2	7	cg24087853 cg27204641 cg07621803 cg05116220 cg03938871 cg25720825 cg07091793 cg02354658 cg01995927 cg15999041	C1orf65 SPIRE2	WZ
clinical AD progression, conversion to MCI or AD	N	34654479	Association of peripheral blood DNA methylation level with Alzheimer's disease progression	Li	2021	Clin Epigenetics	Table S1	1	cg16543836,cg09422696,cg04240108		HH
clinical AD progression, conversion to MCI or AD	N	34654479	Association of peripheral blood DNA methylation level with Alzheimer's disease progression	Li	2021	Clin Epigenetics	Table S1	1	cg00386386, cg09422696, cg00386386,cg09422696, cg27291238 cg23419606 cg03770210 cg09596844 cg16543836 cg08371034 cg17815933 cg15575375 cg04006352 cg11344362 cg12095167 cg13520520 cg12112853 cg00333598	FOXJ2	Ddos
clinical AD progression, conversion to MCI or AD	N	34654479	Association of peripheral blood DNA methylation level with Alzheimer's disease progression	Li	2021	Clin Epigenetics	Table S1	1	cg18555909 cg22765626	HOXA4 CYBC1	WZ
clinical AD diagnosis, conversion to MCI	N	32745807	An epigenome-wide association study of Alzheimer's disease blood highlights robust DNA hypermethylation in the HOXB6 gene	Roubroeks	2020	Neurobiol Aging	Table 2<br>Supp Table 1-5	6	cg13253856,cg00148825,cg03075736,cg16017904,cg10802700	chr17:46681111-46682414; HOXB-AS3	HH
clinical AD diagnosis, conversion to MCI	N	32745807	An epigenome-wide association study of Alzheimer's disease blood highlights robust DNA hypermethylation in the HOXB6 gene	Roubroeks	2020	Neurobiol Aging	Table 2<br>Supp Table 1-5	6	cg07850832, cg04664179, cg17196805 cg16723002	chr17:46681111–46682414; HOXB6	dos
clinical AD diagnosis, conversion to MCI	N	32745807	An epigenome-wide association study of Alzheimer's disease blood highlights robust DNA hypermethylation in the HOXB6 gene	Roubroeks	2020	Neurobiol Aging	Table 2<br>Supp Table 1-5	6	cg16135995 cg00114478 cg21565421 cg24760753 cg26146855	CPT1B TMEM184A	WZ
dementia diagnosis	N	32671182	Blood DNA methylation signatures to detect dementia prior to overt clinical symptoms	Fransquet	2020	Alzheimers Dement	Table 2<br>Supp Table 3-4	3	cg13616097,cg22022881,cg21048159		HH
dementia diagnosis	N	32671182	Blood DNA methylation signatures to detect dementia prior to overt clinical symptoms	Fransquet	2020	Alzheimers Dement	Table 2<br>Supp Table 3-4	3	cg16274893, cg22871559, cg19217964, cg15197125, cg17750831, cg13153264	chr1: 225117076-225117676	dos
dementia diagnosis	N	32671182	Blood DNA methylation signatures to detect dementia prior to overt clinical symptoms	Fransquet	2020	Alzheimers Dement	Table 2<br>Supp Table 3-4	3	cg01404610 cg12679980 cg22871559		WZ
aging, sex	Y	31892350	An epigenome-wide association study of sex-specific chronological ageing	McCartney	2020	Genome Med.	Supp Table 1-5	5	cg12841266,cg13466600,cg09725915,cg15148145,cg24401557		HH
aging, sex	Y	31892350	An epigenome-wide association study of sex-specific chronological ageing	McCartney	2020	Genome Med.	Supp Table 1-5	5	cg16867657, cg24724428, cg06784991	FIGN (sup table 4)	dos
aging, sex	Y	31892350	An epigenome-wide association study of sex-specific chronological ageing	McCartney	2020	Genome Med.	Supp Table 1-5	5	cg16867657 cg24724428 cg12841266 cg21572722 cg17110586		WZ
aging	N	32147700	Blood DNA Methylation and Aging: A Cross-Sectional Analysis and Longitudinal Validation in the InCHIANTI Study	Tharakan	2020	J Gerontol A Biol Sci Med Sci	Table 1<br>Supp Table 2	2	cg02228185,cg24724428	chr1:207997020-207997020<br>chr6:11044877-11044888	HH
aging	N	32147700	Blood DNA Methylation and Aging: A Cross-Sectional Analysis and Longitudinal Validation in the InCHIANTI Study	Tharakan	2020	J Gerontol A Biol Sci Med Sci	Table 1<br>Supp Table 2	2	cg02228185 cg02519751 cg04875128 cg10501210 cg12317815 cg24724428	ELOVL2	WZ
aging	N	25404168	Age-related variations in the methylome associated with gene expression in human monocytes and T cells	Reynolds	2014	Nat Commun	Supp Data 1	1	cg00039326,cg00158333,cg00390775		HH
aging	N	25404168	Age-related variations in the methylome associated with gene expression in human monocytes and T cells	Reynolds	2014	Nat Commun	Supp Data 1	1	cg00004667 cg00006081 cg00008033 cg00015319 cg00032805		WZ
aging	N	25701668	A meta-analysis on age-associated changes in blood DNA methylation: results from an original analysis pipeline for Infinium 450k data	Bacalini	2015	Aging	Table 1	1		ABHD14A, AKAP8L (and their regions)	HH
aging	N	25701668	A meta-analysis on age-associated changes in blood DNA methylation: results from an original analysis pipeline for Infinium 450k data	Bacalini	2015	Aging	Table 1	1		ABHD14A AMER3	WZ
aging, sex	Y	33276343	Age-related DNA methylation changes are sex-specific: a comprehensive assessment	Yusipov	2020	Aging	Supp Table 1, 3	2	cg17077610,cg04891961,cg10546176,cg16383222,cg19458410,cg26227957		HH
aging, sex	Y	33276343	Age-related DNA methylation changes are sex-specific: a comprehensive assessment	Yusipov	2020	Aging	Supp File 1, 3	2	cg01620164 cg03890691 cg23626733 cg23928726 cg27615582		WZ
aging, sex	N	30264654	Epigenetic influences on aging: a longitudinal genome-wide methylation study in old Swedish twins	Wang	2018	Epigenetics	Supp Table 1	1	cg07547549,cg05304393,cg27401724		HH

aging, sex	N	30264654	Epigenetic influences on aging: a longitudinal genome-wide methylation study in old Swedish twins	Wang	2018	Epigenetics	Supp Table 1	1	cg16867657 cg22454769 cg10917602		WZ
aging, sex	N	30264654	Epigenetic influences on aging: a longitudinal genome-wide methylation study in old Swedish twins	Wang	2018	Epigenetics	Supp Table 1	1	cg16867657 cg22454769 cg10917602		WZ
aging, sex	N	25888029	Ageing-associated changes in the human DNA methylome: genomic locations and effects on gene expression	Marttila	2015	BMC Genomics	Supp 1, 9	2	cg15416179,cg00552235		HH
aging, sex	N	25888029	Ageing-associated changes in the human DNA methylome: genomic locations and effects on gene expression	Marttila	2015	BMC Genomics	Supp 1, 9	2	cg16867657 cg16762684 cg11344352 cg17110586 cg04875128		WZ
biomarker	N	37038196	Distinct CSF biomarker-associated DNA methylation in Alzheimer's disease and cognitively normal subjects	Zhang	2023	Alzheimers Res. Ther.	Table 2-5<br>Supp Table 4-9	10	cg04608146,cg09379609,cg05020081,cg01784297,cg25912009,cg27305835,cg26510017,cg27658391,cg07880109,cg11585156	chr3:24536252-24537408; THRB<br>chr15:91473059-91473570; UNC45A,HDDC3<br>chr7:27183946-27184668; HOXA5<br>chr12:39299326-39299727; CPNE8	HH
biomarker	N	37038196	Distinct CSF biomarker-associated DNA methylation in Alzheimer's disease and cognitively normal subjects	Zhang	2023	Alzheimers Res. Ther.	Table 2-5<br>Supp Table 4-9	10	cg01373819 cg10961330 cg07755173 cg04608146 cg21481456	TNFRSF11A UNC45A	WZ
aging, epignetic clock	N	32367804	PoAM	Belsky	2020	Elife	Supp File 1A	1	cg26981978; cg27165794;cg24125710;cg00668559 (checked betas associated) checked total numbe rof Cpgs		dos
aging, epignetic clock	N	32367804	PoAM	Belsky	2020	Elife	Supp File 1A	1	Full table		HH
aging, epignetic clock	N	32367804	PoAM	Belsky	2020	Elife	Supp File 1A	1	Full table		WZ
aging, epignetic clock	N	30048243	Epigenetic clock for skin and blood cells applied to Hutchinson Gilford Progeria Syndrome and ex vivo studies	Horvath	2018	Aging	Supp Data 2	1	Full table		HH
aging, epignetic clock	N	30048243	Epigenetic clock for skin and blood cells applied to Hutchinson Gilford Progeria Syndrome and ex vivo studies	Horvath	2018	Aging	Supp Data 2	1	cg00503840;cg27544190		dos
aging, epignetic clock	N	30048243	Epigenetic clock for skin and blood cells applied to Hutchinson Gilford Progeria Syndrome and ex vivo studies	Horvath	2018	Aging	Supp Data 2	1	Full table		WZ
aging, epignetic clock	N	23177740	Genome-wide Methylation Profiles Reveal Quantitative Views of Human Aging Rates	Hannum	2013	Mol. Cell.	Supp Table 2	1	Full table		HH
aging, epignetic clock	N	23177740	Genome-wide Methylation Profiles Reveal Quantitative Views of Human Aging Rates	Hannum	2013	Mol. Cell.	Supp Table 2	1	cg03399905;cg03032497;cg25478614		
aging, epignetic clock	N	23177740	Genome-wide Methylation Profiles Reveal Quantitative Views of Human Aging Rates	Hannum	2013	Mol. Cell.	Supp Table 2	1	Full table		dos
aging, epignetic clock	N	24138928	DNA methylation age of human tissues and cell types	Horvath	2013	Genome Biol.	Supp File 3	1	Full table		HH
aging, epignetic clock	N	24138928	DNA methylation age of human tissues and cell types	Horvath	2013	Genome Biol.	Supp File 3	1	cg00075667;cg00091693;cg02479575;cg27544190;cg27413543		dos
aging, epignetic clock	N	24138928	DNA methylation age of human tissues and cell types	Horvath	2013	Genome Biol.	Supp File 3	1	full table		dos
aging, epignetic clock	N	24138928	DNA methylation age of human tissues and cell types	Horvath	2013	Genome Biol.	Supp File 3	1	Full table		WZ
aging, epignetic clock	N	21731603	Epigenetic predictor of age	Bocklandt	2011	PLoS One	Supp Table 1-2	2	Full table		HH
aging, epignetic clock	N	21731603	Epigenetic predictor of age	Bocklandt	2011	PLoS One	Supp Table 1-2	2	cg02154186;cg02228185;cg25511429;cg12799895		dos
aging, epignetic clock	N	21731603	Epigenetic predictor of age	Bocklandt	2011	PLoS One	Supp Table 1-2	2	Full table		WZ
aging, epignetic clock	N	29676998	An epigenetic biomarker of aging for lifespan and healthspan	Levine	2018	Aging	Supp File 2	1	Full table		HH
aging, epignetic clock	N	29676998	An epigenetic biomarker of aging for lifespan and healthspan	Levine	2018	Aging	Supp File 2	1	Full table		dos
aging, epignetic clock	N	29676998	An epigenetic biomarker of aging for lifespan and healthspan	Levine	2018	Aging	Supp File 2	1	Full table		WZ
aging, epignetic clock	N	33707346	Development of the VISAGE enhanced tool and statistical models for epigenetic age estimation in blood, buccal cells and bones	Woźniak	2021	Aging	Supp Table 1	1	Full table		HH
aging, epignetic clock	N	33978960	Genome-wide identification of age-related CpG sites for age estimation from blood DNA of Han Chinese individuals	Xiao	2021	Aging	Supp Table 3-5	3	Full table		WZ
aging, epignetic clock	N	33978960	Genome-wide identification of age-related CpG sites for age estimation from blood DNA of Han Chinese individuals	Xiao	2021	Aging	Supp Table 3-5	3	Full table		HH
aging, epignetic clock	N	33978960	Genome-wide identification of age-related CpG sites for age estimation from blood DNA of Han Chinese individuals	Xiao	2021	Aging	Supp Table 3-5	3	Full table		dos
aging, epignetic clock	N	33978960	Genome-wide identification of age-related CpG sites for age estimation from blood DNA of Han Chinese individuals	Xiao	2021	Aging	Supp Table 3-5	3	Full table		WZ
aging, epignetic clock	N	35029144	DunedinPACE, a DNA methylation biomarker of the pace of aging	Belsky	2022	Elife	Supp File 1B	1	Full table		HH

aging, epignetic clock	N	35029144	DunedinPACE, a DNA methylation biomarker of the pace of aging	Belsky	2022	Elife	Supp File 1B	1	cg269819778; cg27165794;cg24125710;cg00668559		dos
aging, epignetic clock	N	35029144	DunedinPACE, a DNA methylation biomarker of the pace of aging	Belsky	2022	Elife	Supp File 1B	1	Full table		WZ
aging, epignetic clock	N	31443728	Improved precision of epigenetic clock estimates across tissues and its implication for biological ageing	Zhang	2019	Genome Med.	Github[1]	1	Full table		HH
aging, epignetic clock	N	31443728	Improved precision of epigenetic clock estimates across tissues and its implication for biological ageing	Zhang	2019	Genome Med.	Github[1]	1	Full table		
aging, epignetic clock	N	31443728	Improved precision of epigenetic clock estimates across tissues and its implication for biological ageing	Zhang	2019	Genome Med.	Github[1]	1	cg01612140;cg05575921;cg23605802		dos
aging, epignetic clock	N	26928272	life expectancy and age	Lin	2016	Aging	Table 2	1	cg00059225;cg02844545;cg15379633;cg26610806		dos
aging, epignetic clock	N	33300551	cortical clock	Shirby	2020	Brain	Github[2]	1	cg0059225;cg00771642;cg00924265;cg03594801;cg15540623		dos
aging, epignetic clock	N	27471517	Vidal_bralo	Vidal Bralo	2016	Frontiers in genetics	Table 2	1			dos
AD neuropathology	N	33257653	Epigenome-wide meta-analysis of DNA methylation differences in prefrontal cortex implicates the immune processes in Alzheimer's disease	Zhang	2020	Nature Communication	Supp data 15, Supp data 2	2		chr19:49220102-49220485<br>HOXA2	WZ
AD neuropathology	N	33257653	Epigenome-wide meta-analysis of DNA methylation differences in prefrontal cortex implicates the immune processes in Alzheimer's disease	Zhang	2020	Nature Communication	Supp data 15, Supp data 2	2	cg04917446, cg14103343, cg01111041, cg07328519, cg01027532	chr7:98739496-98739782<br>AZU1	HH
AD neuropathology	Y	33902726	Sex-specific DNA methylation changes in Alzheimer's disease pathology	Zhang	2021	Acta Neuropathologica Communications	Supp Table 3-6	4	cg21806242<br>cg05235171<br>cg07061298<br>cg13390284<br>cg05731218<br>cg06635946	chr7:27153580-27153944<br>CDH9	WZ
AD neuropathology	Y	33902726	Sex-specific DNA methylation changes in Alzheimer's disease pathology	Zhang	2021	Acta Neuropathologica Communications	Supp Table 3-6	4	cg15610437, cg12307200, cg17881200;<br>cg15033653, cg04917446, cg06119452	chr15:40268421-40268777<br>TRIO	HH
AD neuropathology	N	25129075	Alzheimer's disease: early alterations in brain DNA methylation at ANK1, BIN1, RHBDF2 and other loci.	De Jager	2014	Nat Neuroscience	Supplementary Table 2	1	cg11724984 cg23968456 cg15821544		WZ
AD neuropathology	N	25129075	Alzheimer's disease: early alterations in brain DNA methylation at ANK1, BIN1, RHBDF2 and other loci.	De Jager	2014	Nat Neuroscience	Supplementary Table 2	1	cg11724984, cg00621289, cg21207436		HH
AD neuropathology	N	25129077	Methylomic profiling implicates cortical deregulation of ANK1 in Alzheimer's disease	Lunnon	2014	Nat Neuroscience	Supp Table 3-6, 12	5	cg04525464<br>cg06108383<br>cg10752406<br>cg25018458<br>cg13942103<br>cg00767503<br>cg04147621 cg05726109 cg11724984		WZ
AD neuropathology	N	25129077	Methylomic profiling implicates cortical deregulation of ANK1 in Alzheimer's disease	Lunnon	2014	Nat Neuroscience	Supp Table 3-6, 12	5	cg24770624, cg02672452;<br>cg25018458, cg02961798;<br>cg15928398, cg21966754;<br>cg20767910, cg25590527;<br>cg17074958, cg03308986		HH
AD neuropathology	N	34112773	A meta-analysis of epigenome-wide association studies in Alzheimer's disease highlights novel differentially methylated loci across cortex	Smith	2021	Nature Communication	Supp Data 1-6	6	cg22962123 cg21806242 cg07061298 cg01301319 cg05731218<br>cg11823178 cg05417607 cg25018458 cg02674693 cg05066959	chr7:27153212-27155234<br>chr19:10736006-10736448<br>chr7:27146237-27146445<br>chr19:49220102-49220235<br>ANK1	WZ
AD neuropathology	N	34112773	A meta-analysis of epigenome-wide association studies in Alzheimer's disease highlights novel differentially methylated loci across cortex	Smith	2021	Nature Communication	Supp Data 1-6	6	cg02317313, cg09144964;<br>cg05417607, cg04658679;<br>cg20618448, cg25018458	chr19:39798481-39799137<br>chr15:93617030-93617168<br>CMYA5	HH
AD neuropathology	N	36811307	Genome-wide methylomic regulation of multiscale gene networks in Alzheimer's disease	Wang	2023	Alzheimers Dement.	Supp data S2a S2c	2	cg00001687<br>cg00005437<br>cg00009919<br>cg00010900	chr1:1022900-1023320	WZ
AD neuropathology	N	36811307	Genome-wide methylomic regulation of multiscale gene networks in Alzheimer's disease	Wang	2023	Alzheimers Dement.	Supp data S2a S2c	2	cg00026363, cg00032703, cg00037314	chr1:1974661-1974942	HH
AD neuropathology	N	29550519	Elevated DNA methylationacross a 48-kb region spanning the HOXAgene cluster is associated with Alzheimer'sdisease neuropathology.	Smith	2018	Alzheimers & Dementia	Supp Table 2 Table 2	2	cg22867816<br>cg06977285<br>cg05783384<br>cg07349815	C11orf21<br>chr11:2321770-2323247	WZ
AD neuropathology	N	29550519	Elevated DNA methylationacross a 48-kb region spanning the HOXAgene cluster is associated with Alzheimer'sdisease neuropathology.	Smith	2018	Alzheimers & Dementia	Supp Table 2 Table 2	2	cg13714797, cg17067993	chr7:27154262-27155234<br>STOX2	HH

AD neuropathology	N	33790779	A Meta-Analysis of Brain DNA Methylation Across Sex, Age, and Alzheimer's Disease Points for Accelerated Epigenetic Aging in Neurodegeneration	Pellegrini	2021	Front. Aging Neurosci.	Supp File 1 3 5 6	4	cg04672450<br>cg05527507<br>cg10176463<br>cg21139076<br>cg07549208<br>cg13904806 cg23731742 cg06615380 cg23629166<br>cg06425162<br>cg14505174<br>cg08883134<br>cg06101225		WZ
AD neuropathology	N	33790779	A Meta-Analysis of Brain DNA Methylation Across Sex, Age, and Alzheimer's Disease Points for Accelerated Epigenetic Aging in Neurodegeneration	Pellegrini	2021	Front. Aging Neurosci.	Supp File 1 3 5 6	4	cg14488957, cg12625874, cg12909536;<br>cg05392440, cg05303132, cg17001566;<br>cg09332175, cg16318112, cg18172484;<br>cg18024260, cg14288281, cg13560058;<br>cg22718050, cg02577049, cg05355142;		HH
Sex	Y	30976086	Sex-differential DNA methylationand associated regulation networks in humanbrain implicated in the sex-biased risks ofpsychiatric disorders.	Xia	2021	Moleular psychiatry	Supp Table 2-3	2	cg20332503<br>cg20818151<br>cg04524851<br>cg01860821<br>cg24826745	chr1:894207-895413<br>chr1:1168432-1168550<br>KLHL17<br>AGRN	
Sex	Y	30976086	Sex-differential DNA methylationand associated regulation networks in humanbrain implicated in the sex-biased risks ofpsychiatric disorders.	Xia	2021	Moleular psychiatry	Supp Table 2-3	2	cg24826745, cg14742809, cg19602446	chr1:154841980-154842862<br>SHC1	HH
AD neuropathology; Dementia	N	37149695	Entorhinal cortex epigenome-wide associationstudy highlights four novel loci showingdifferential methylation in Alzheimer's disease	Sommerer	2023	Alzheimers Res. Ther.	external website[3]	2	cg21036164<br>cg00325458<br>cg06300892<br>cg16464569<br>cg22090150<br>cg25191519<br>cg25436217<br>cg03258927<br>cg19627207<br>cg23237276<br>cg16464569<br>cg27648238		
AD neuropathology; Dementia	N	37149695	Entorhinal cortex epigenome-wide associationstudy highlights four novel loci showingdifferential methylation in Alzheimer's disease	Sommerer	2023	Alzheimers Res. Ther.	external website[3]	2	cg27648238, cg14598211;<br>cg25191519, cg18748888		HH
AD neuropathology	N	33069246	Epigenome-wide association study of Alzheimer's disease replicates 22 differentially methylated positions and 30 differentially methylated regions.	Li	2020	Clin Epigenetics	Supp1 Supp 2A-B	3	cg26263477<br>cg26210521<br>cg13388025<br>cg20396870<br>cg14453612<br>cg14058329<br>cg13375849<br>cg26796807<br>cg09448088	TFAP2E<br>chr1:55247140-55247408	
AD neuropathology	N	33069246	Epigenome-wide association study of Alzheimer's disease replicates 22 differentially methylated positions and 30 differentially methylated regions.	Li	2020	Clin Epigenetics	Supp1 Supp 2A-B	3	cg00565657, cg26541517, cg16893790	chr10:73521606-73521755<br>CD82	HH
AD neuropathology; Sex	N	36596913	Integrated DNA Methylation/RNA Profiling in Middle Temporal Gyrus of Alzheimer's Disease	Piras	2023	Cell Mol Neurobio.	Supp Table 1 3 7 9 10	5	cg11823178<br>cg05066959<br>cg05313153<br>cg17693222<br>cg12307200<br>cg09109520<br>cg00170188<br>cg00400340<br>cg00503690<br>cg01522592<br>cg01753263<br>cg01999701	PRDM16<br>chr13:113698408-113700027	
AD neuropathology; Sex	N	36596913	Integrated DNA Methylation/RNA Profiling in Middle Temporal Gyrus of Alzheimer's Disease	Piras	2023	Cell Mol Neurobio.	Supp Table 1 3 7 9 10	5	cg02158822, cg02295973, cg03072035;<br>cg20942162, cg20865082	chr5:151066268-151067341<br>NKD2	HH

[1] https://github.com/qzhang314/DNAm-based-age-predictor/blob/master/en.coef
[2] https://github.com/gemmashireby/CorticalClock
[3] https://www.liga.uni-luebeck.de/sommerer_AD_entorhinal_cortex_EWAS_results/
